# Artificial intelligence in musculoskeletal radiology: practical aspects and latest perspectives

**DOI:** 10.1093/bjro/tzaf029

**Published:** 2025-11-09

**Authors:** Mickael Tordjman, Jan Fritz, Nor-Eddine Regnard, Richard Kijowski, Fadila Mihoubi, Bachir Taouli, Xueyan Mei, Mingqian Huang, Ali Guermazi

**Affiliations:** Department of Radiology, Biomedical Engineering and Imaging Institute, Icahn School of Medicine at Mount Sinai, New York, NY 10029, United States; Department of Radiology, New York University Grossman School of Medicine, New York, NY 10016, United States; Gleamer, Paris, 75010, France; Réseau d‘Imagerie Sud Francilien, Clinique du Mousseau Ramsay Santé, Evry, 91042, France; Pôle Médical Sénart, Lieusaint, 77127, France; Department of Radiology, New York University Grossman School of Medicine, New York, NY 10016, United States; Department of Radiology, Cochin Hospital, APHP, Paris, 75014, France; Leonard de Vinci Center, Paris, 75016, France; Department of Radiology, Biomedical Engineering and Imaging Institute, Icahn School of Medicine at Mount Sinai, New York, NY 10029, United States; Department of Radiology, Biomedical Engineering and Imaging Institute, Icahn School of Medicine at Mount Sinai, New York, NY 10029, United States; Department of Radiology, Biomedical Engineering and Imaging Institute, Icahn School of Medicine at Mount Sinai, New York, NY 10029, United States; Department of Radiology, Boston VA Healthcare System, West Roxbury, MA 02132, United States

**Keywords:** artificial intelligence, musculoskeletal, fracture, MRI

## Abstract

Musculoskeletal (MSK) imaging was among the first radiology subspecialties to adopt artificial intelligence (AI), with applications now spanning the entire MSK workflow, from image acquisition to reporting. Deep learning-based reconstruction protocols can accelerate MRI by reducing scan times and artefacts, improving accessibility in high-volume and resource-limited settings. Furthermore, AI interpretation tools have demonstrated strong performance in fracture detection, assessment of meniscal and ligament tears, bone tumour characterization and automated quantification of measurements, supporting greater diagnostic consistency across radiologists with varying experience levels. Large language models (LLMs) extend AI’s impact beyond image analysis by simplifying reports for patients, automating classification systems, and streamlining clinical communication. Despite these advances, important challenges remain. Integration of AI into already established clinical workflows can be complex, and requires robust technical solutions, regulatory compliance, and strategies to maintain radiologist oversight. Questions of liability, cost-effectiveness, and the role of AI in medical education further underscore the need for careful implementation. AI is poised to fundamentally reshape MSK radiology by enhancing efficiency, improving diagnostic accuracy, and enabling more patient-centred communication. To fully realize this potential, adoption must balance innovation with safety, equity, and sustainability, ensuring AI remains a trusted assistive tool that strengthens rather than replaces radiologist expertise.

## Introduction

Artificial intelligence (AI) applications in radiology have expanded rapidly in recent years and musculoskeletal (MSK) imaging was among the earliest radiology subspecialties to explore these innovations. This early adoption likely reflects the broad clinical spectrum of MSK practice, including mechanical disorders, sports-related injuries (including meniscal tears and ligamentous injuries), soft-tissue and bone tumours, and rheumatologic diseases, coupled with the large volume of imaging studies performed. Early AI tools in MSK radiology primarily targeted automated fracture detection,^1^ at first in the appendicular skeleton, and optimization of imaging protocols, aiming to accelerate acquisition and improve image quality, for example in knee and shoulder MRI.^2^ Since then, a wide range of applications have emerged, such as deep learning (DL)-based reconstruction, automated classification of bone and soft-tissue lesions, and AI-assisted interpretation of MRI and radiographs.^3–6^

The current landscape demonstrates clear progress, yet also highlights challenges. Integrating AI into established workflows can be complex, requiring radiologists to trust and validate algorithm outputs while navigating medico-legal and regulatory frameworks. Moreover, the rapid rise of large language models (LLMs) adds a new dimension by extending AI’s role from image analysis to communication, reporting, and education.^7^

This scoping review synthesizes the latest practical applications and perspectives of AI in MSK radiology. Rather than providing isolated summaries, we aim to highlight the key implications of each thematic area, accelerated image acquisition, image interpretation, LLM applications, workflow integration, cost-effectiveness, liability, and education, and demonstrate how these advances collectively reshape MSK radiology in an AI-enabled environment.

## Accelerated image acquisition and improved image quality

**Figure 1.**
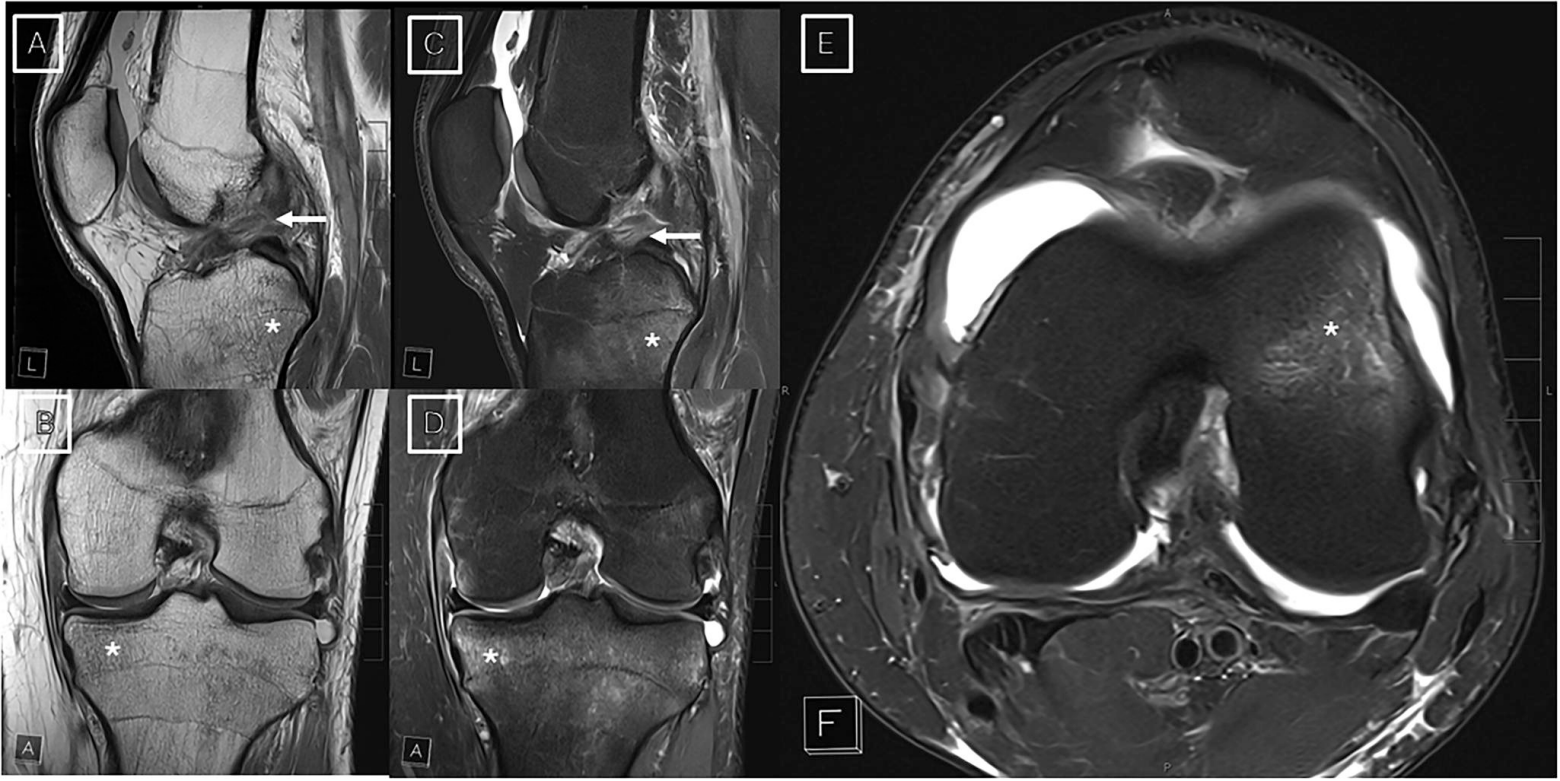
MRI scans in a 23-year-old man with recent knee injury, demonstrating ACL tear (arrows) and bone contusions (stars). Combined threefold parallel imaging (PIx3) and 2-fold simultaneous multislice (SMSx2) accelerated deep learning super-resolution turbo spin-echo sequences (A-E) were acquired at 3 T.

**Figure 2.**
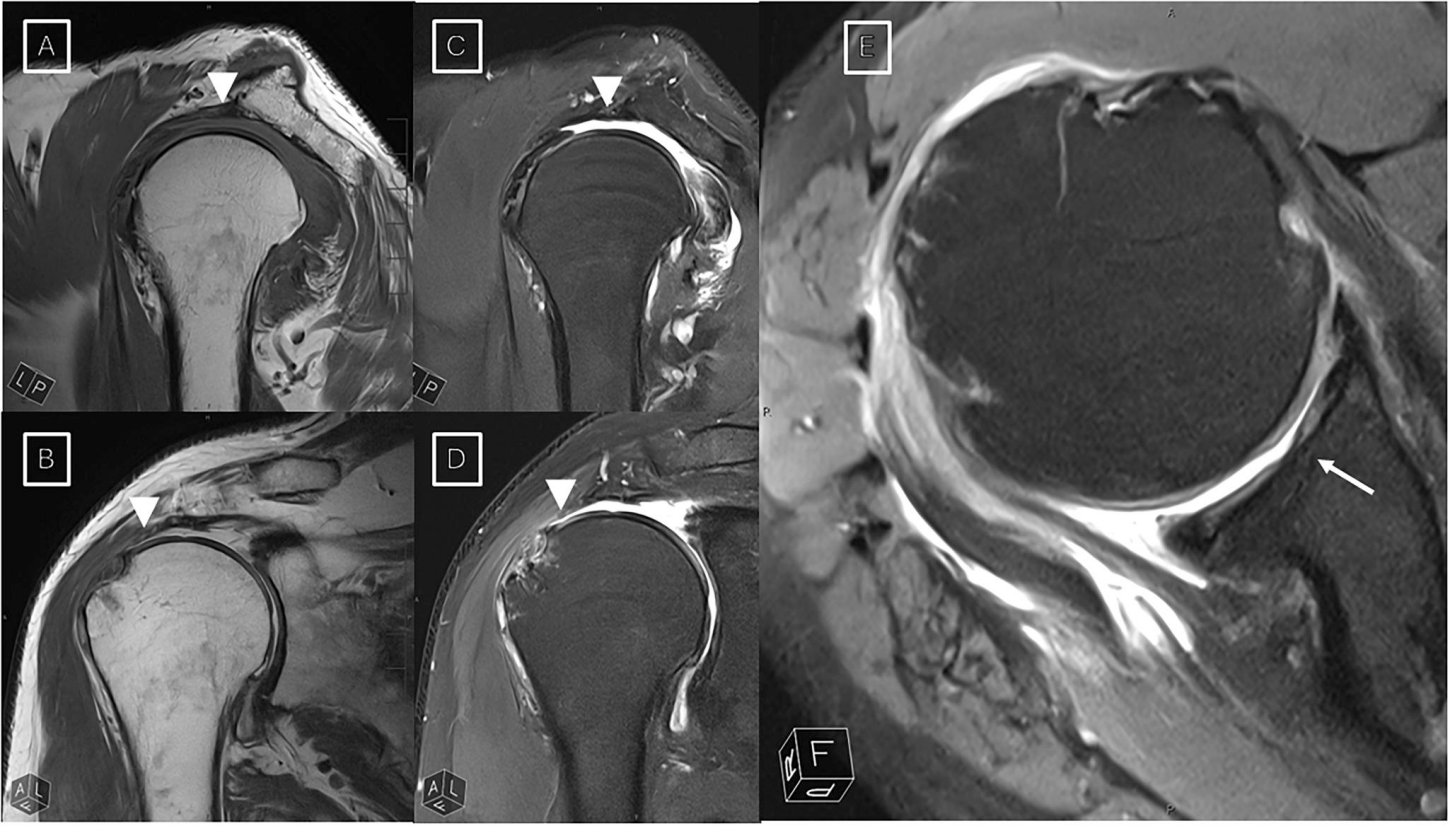
MRI scans in a 77-year-old man with right shoulder pain, demonstrating rotator cuff tear (arrowheads) and articular cartilage defects (arrows). The unenhanced coronal (A and B), axial (C), and sagittal (D and E) deep learning super-resolution (DLSR) 3-fold parallel imaging-accelerated turbo spin-echo MRI scans were obtained at 3.0-T field strength.

MSK imaging includes a wide spectrum of examinations for back pain, mechanical disorders, sports imaging, soft-tissue and bone lesions, and rheumatologic diseases, where MRI remains the principal advanced modality. Two long-standing limitations of MRI are prolonged acquisition times and limited system availability, which are interrelated, and the susceptibility of images to motion or metal-related artefacts that can hinder interpretation. Before the advent of DL, acceleration largely relied on parallel imaging and compressed sensing. Parallel imaging exploits multi-channel receiver coils to reconstruct a complete dataset from fewer phase-encoding steps, thereby shortening repetition times while preserving spatial resolution. Compressed sensing further reduces sampling by taking advantage of the inherent sparsity of MR data in a transform domain; an iterative reconstruction then recovers the missing k-space information with minimal artefact. These techniques remain the physical foundation upon which most DL methods build. Recent AI-driven strategies extend these concepts by training neural networks on fully sampled data to reconstruct under-sampled k-space, achieving reductions up to 53% in scan times without compromising diagnostic quality.^8^^,^^9^ In essence, the AI algorithm “learns” to fill in the missing information, enabling faster acquisitions by using fewer data points. This accelerated imaging approach was first tested on the knee, one of the most commonly imaged joints, where DL reconstruction maintained image quality and diagnostic performance comparable to conventional MRI protocols^2^ (Figure 1). In the study by Johnson et al,^2^ radiologists who evaluated these reconstructions reported similar confidence levels in interpreting DL acquisitions compared with conventional reconstructions (deeming these images “clinically interchangeable”) for meniscal and ligament tears, and bone marrow and cartilage abnormalities. Investigators have also applied related DL super-resolution (DLSR) and parallel imaging hybrid approaches to other joints. For example, DLSR-accelerated turbo spin-echo sequences have been used to substantially shorten shoulder MRI examinations while preserving diagnostic accuracy (Figure 2). These results reinforce the broader principle that DL-based reconstruction can generalize across MSK applications, offering meaningful time savings without loss of image quality.

Beyond accelerating acquisition times, AI-based image reconstruction algorithms also improve image sharpness, reduce noise, and correct motion or metallic artefacts.^10^ This is achieved through denoising algorithms, super-resolution techniques, and advanced post-processing frameworks. DL reconstructions often yield higher signal-to-noise ratios and more uniform fat suppression compared with conventional techniques, both of which contribute to clearer delineation of small anatomic structures. These algorithms can also enhance contrast resolution by better distinguishing adjacent soft-tissue structures, allowing clearer visualization of low-contrast lesions such as early marrow oedema. Therefore, these improvements can enhance diagnostic performance. For instance, subtle findings like minor chondral defects, small tendon tears, and subtle bone marrow oedema patterns may be more readily visualized in scans that have been enhanced through AI algorithms due to their improved image quality.^11^^,^^12^ Nevertheless, aggressive under-sampling carries a theoretical risk of information loss, and reports describe missed small lesions in heavily accelerated datasets, underscoring the need for careful protocol optimization.^10^

AI algorithms have already been integrated in most of the novel cross-sectional imaging systems. AI-accelerated acquisition and reconstruction techniques are becoming a new standard, with great impact in high-volume MSK radiology practices.^13^

Key takeaway: AI-driven reconstruction techniques are not only shortening exam times but also improving image quality in ways that enhance diagnostic confidence. For MSK radiology, this means increased throughput in high-volume centres, more consistent detection of subtle pathology, and improved accessibility in regions with limited MRI availability.

## AI for image interpretation

**Figure 3.**
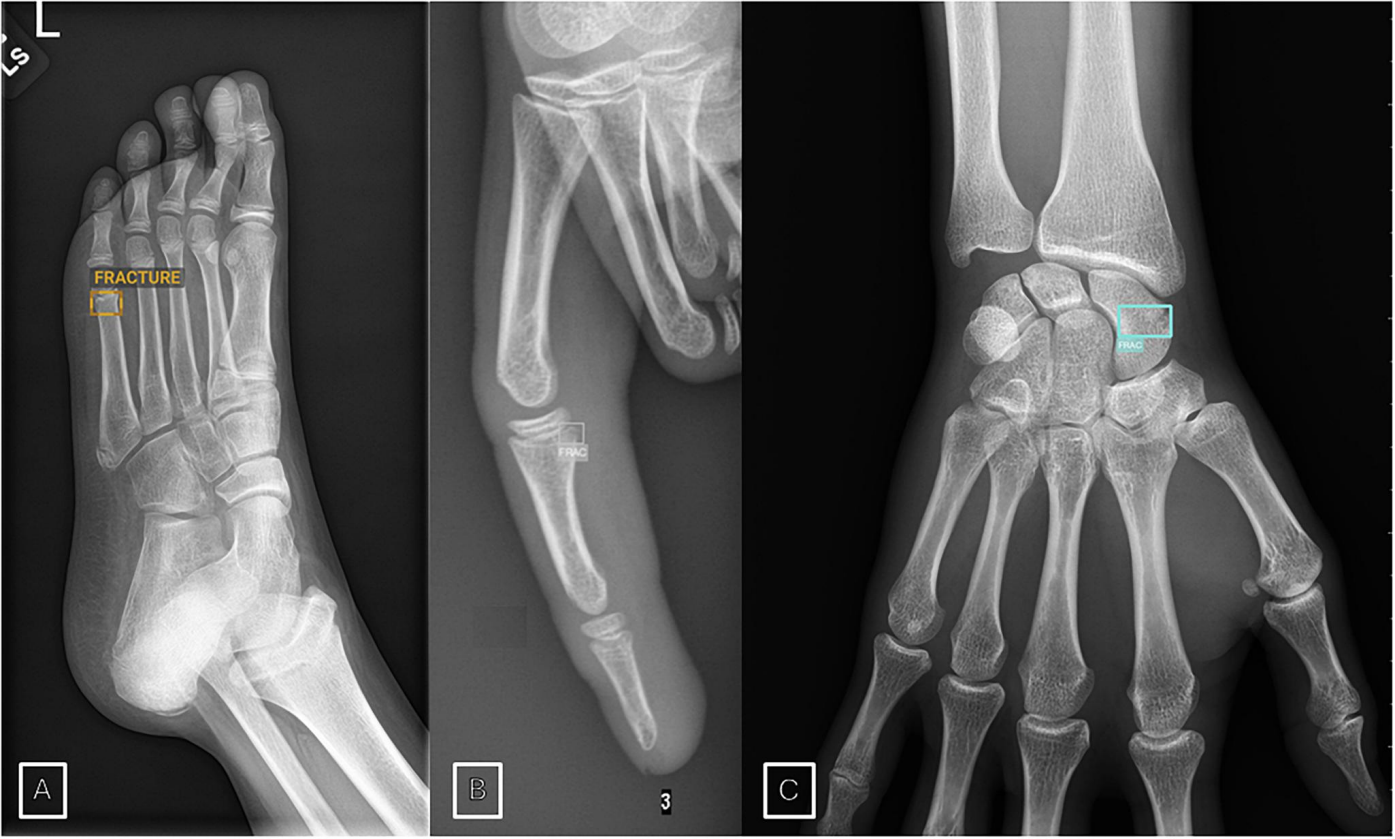
AI detection of subtle fractures. Distal metaphyseal buckle fracture of the fifth metatarsal bone detected on radiograph using AI software (Boneview by GLEAMER) (A). Phalangeal fracture detected by another AI tool (MILVUE Suite 2.4.1) (B). Scaphoid fracture detected using MILVUE Suite 2.4.1 (C).

One of the earliest and most intuitive applications of AI in MSK radiology has been fracture detection on radiographs, with multiple commercial solutions nowadays available and clinically used (see Table 1).^14^ In busy clinical settings such as emergency departments, missed or delayed diagnoses can lead to significant morbidity. Thus, the use of these tools based on convolutional neural networks (CNNs) has demonstrated great potential. The use of AI algorithms for fracture detection was associated with an improved sensitivity and specificity in readers with varying level of expertise, including resident radiologists, emergency physicians and expert MSK radiologists^15^ (Figure 3). Multiple studies demonstrated that the performance of AI-aided radiologist is improved compared to AI alone or radiologist alone.^16^ Furthermore, these tools may increase the interpretation time and potentially decrease emergency room stays.

**Table 1.** Examples of AI tools used in MSK imaging.

Fracture detection	BoneView	Gleamer
	SmartUrgences	Milvue
	Rayvolve	AZMed
	qMSK	Qure.ai
Bone age estimation	BoneXpert	Visiana
	Panda	ImageBiopsy Lab
	BoneAge	VunoMed
Automated measurements	Lama	ImageBiopsy Lab
	BoneMetrics	Gleamer
	SmartXpert	Milvue
	RBknee	Radiobotics

**Figure 4.**
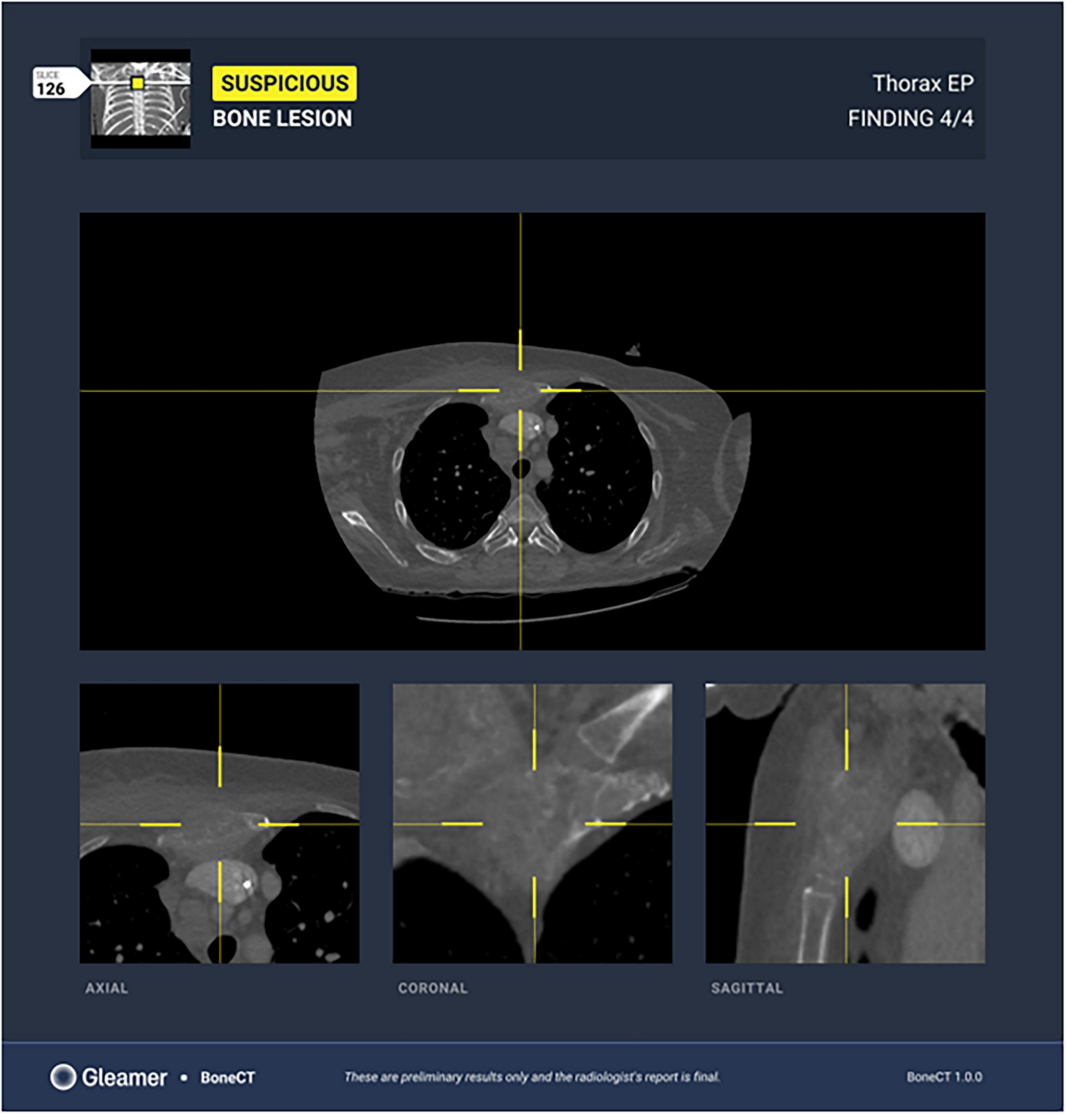
Detection of lytic focal bone lesion of the sternum on CT-scanner with GLEAMER BoneCT software.

Bone tumour detection is another application of AI (Figure 4), currently at the research stage, that could improve the detection and classification of malignant lesions, sometimes difficult to differentiate from benign lesions, especially for non-expert radiologists, based on morphological features, X-rays, CT and MRI patterns.^17^ Additionally, radiomics models have been used to analyse tumour shape, texture, and intensity patterns, to further improve the differentiation between benign lesions and malignant tumours.^18^

Other AI algorithms were trained for meniscal tear detection and classification on knee MRI. In previous research studies, AI-driven models provided classification of meniscal tear, horizontal, radial, vertical, or complex tears, and could even assess meniscal extrusion.^19^^,^^20^ These capabilities could prove valuable for surgical planning and standardization of meniscal tear description. Preliminary results are encouraging, and future large-scale studies are needed to confirm these findings before these tools can be implemented.^21^

Additional possibilities include both anterior and posterior cruciate ligaments (ACL/PCL) evaluation. AI tools that evaluate ACL and PCL tears often involve segmentation algorithms of the ligaments, followed by classification systems that identify partial or complete tears by evaluating the ligament’s thickness, signal intensity, and continuity.^22^

Osteoporosis and osteopenia are very frequent in the general population, in particular in older women, with an important impact on the morbidity and mortality. Opportunistic screening enhanced with AI on CT or radiographs performed for other purposes may contribute to the early detection and treatment of these patients.^23^ Similarly, whole body composition and in particular sarcopenia have been demonstrated to be important prognostic factors of numerous disorders including cancers and chronic inflammatory diseases. AI models have been used for automated segmentation and quantification of the fat and muscles, which could provide valuable metrics for personalized care.^24^ These areas of research may lead to additional clinical available AI software in the future.

**Figure 5.**
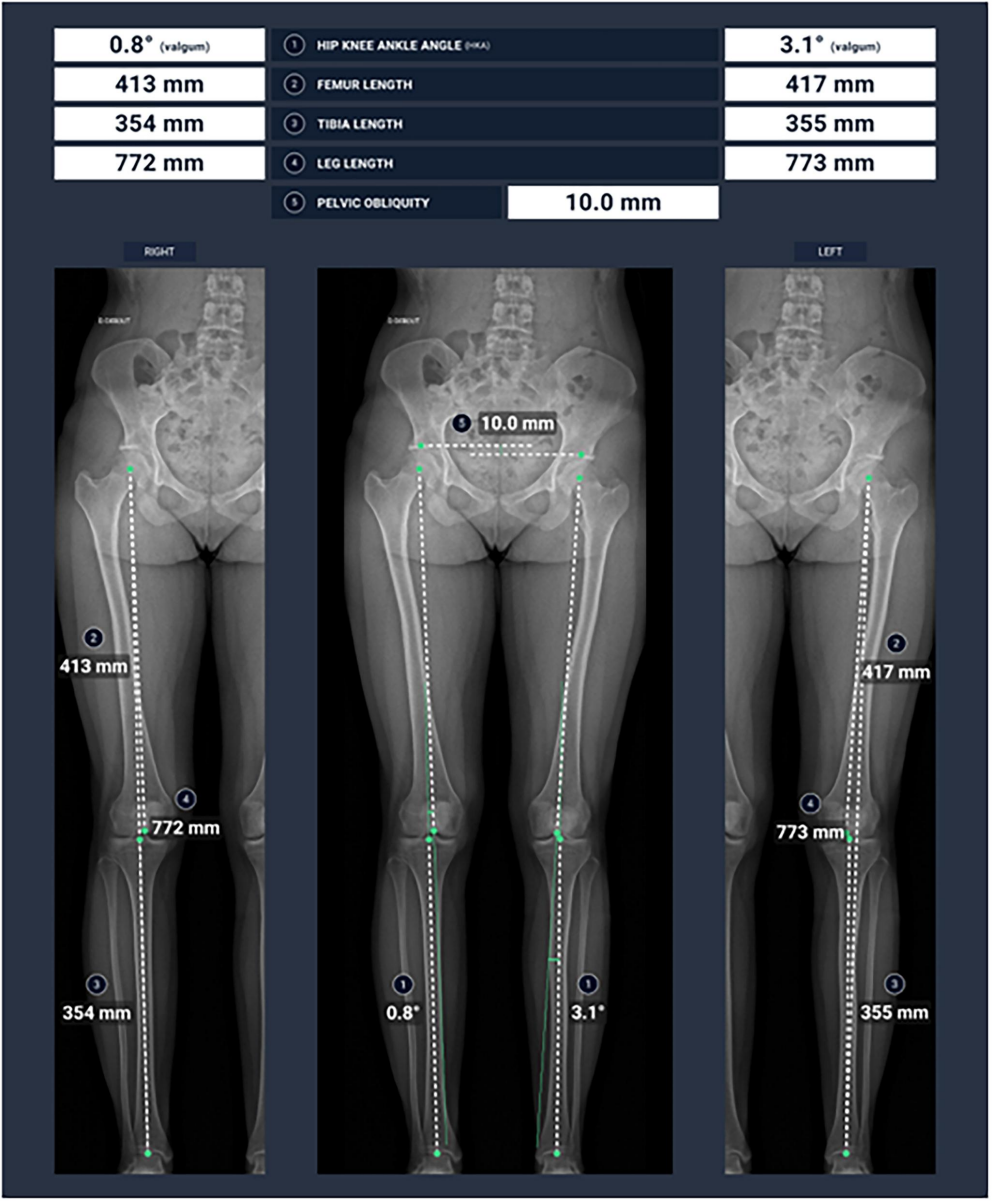
Automatic gonometric measurement of lower limb axes using GLEAMER's Bonemetrics software.

**Figure 6.**
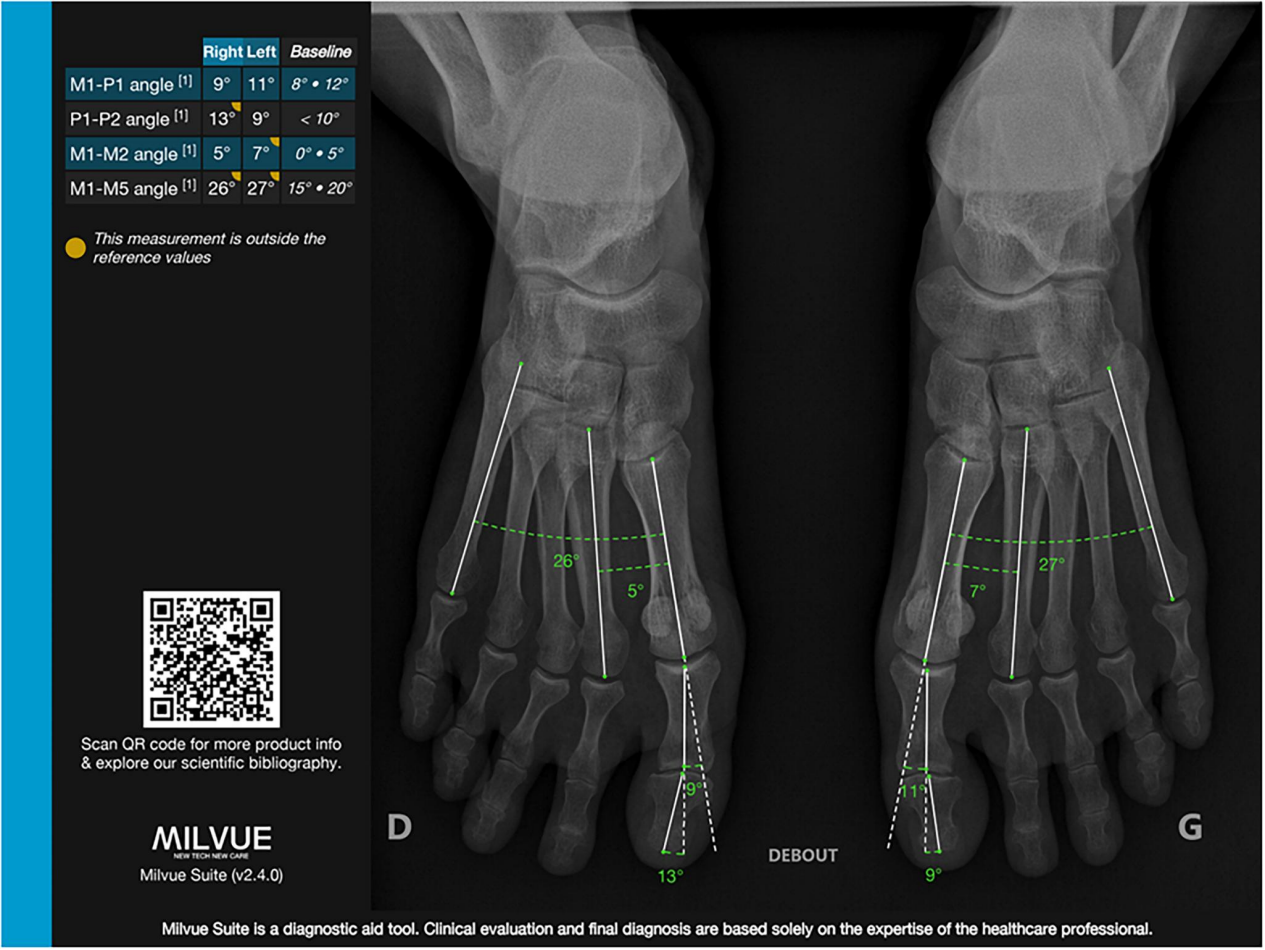
Automated measurements of feet radiographs, Milvue Suite (milvue).

**Figure 7.**
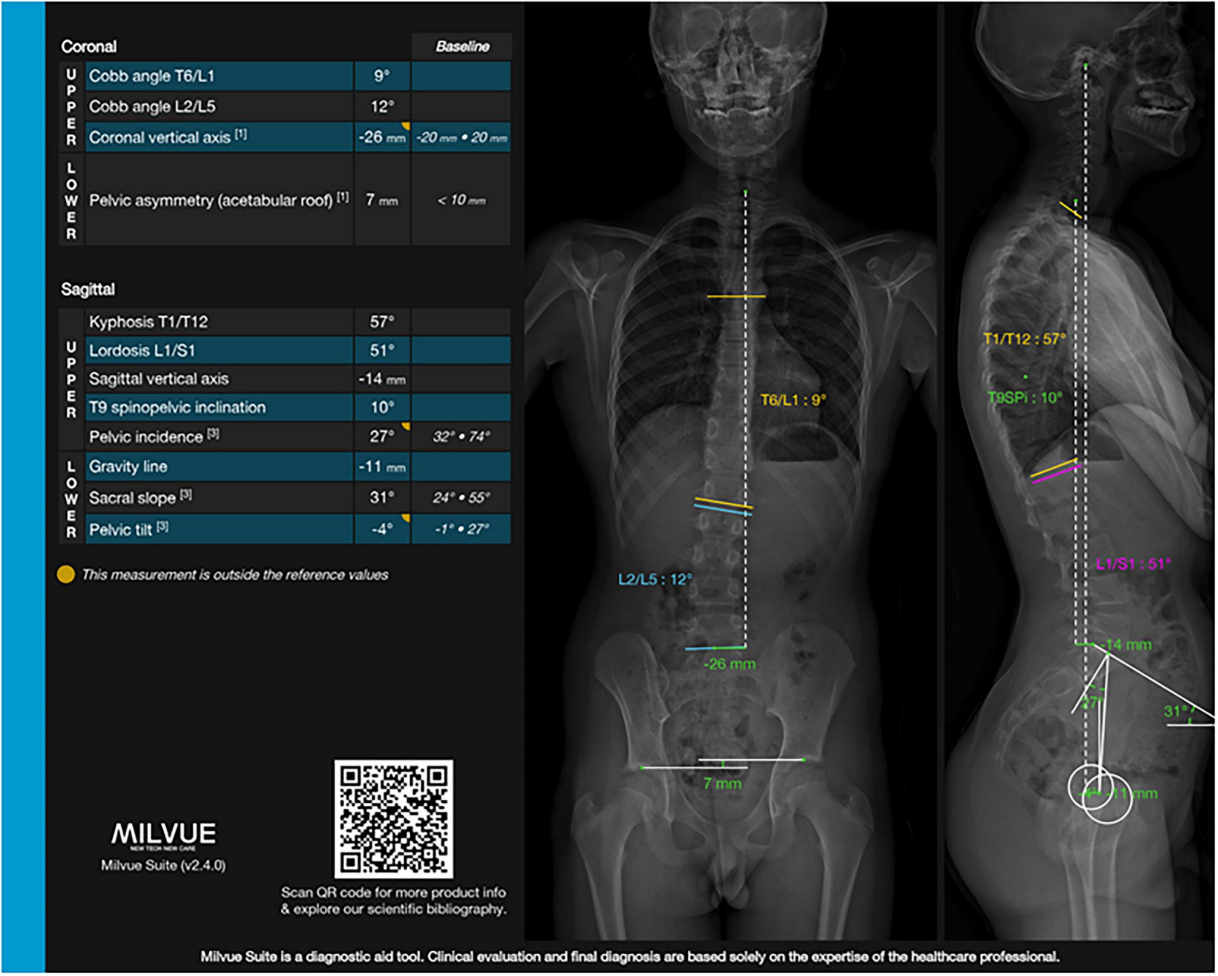
Cobb angle automated measurement for scoliosis (Milvue, Milvue suite).

MSK imaging is associated with multiple measurements on the different modalities which are very time consuming. A few examples include the M1-P1 angle for hallux valgus (Figures 5 and 6), the Cobb angle for scoliosis detection and follow-up (Figure 7) and the different angles used to detect hip dysplasia. Different clinically available tools currently focus on automatizing these measurements, with great time gain for radiologists.^25^^,^^26^

**Figure 8.**
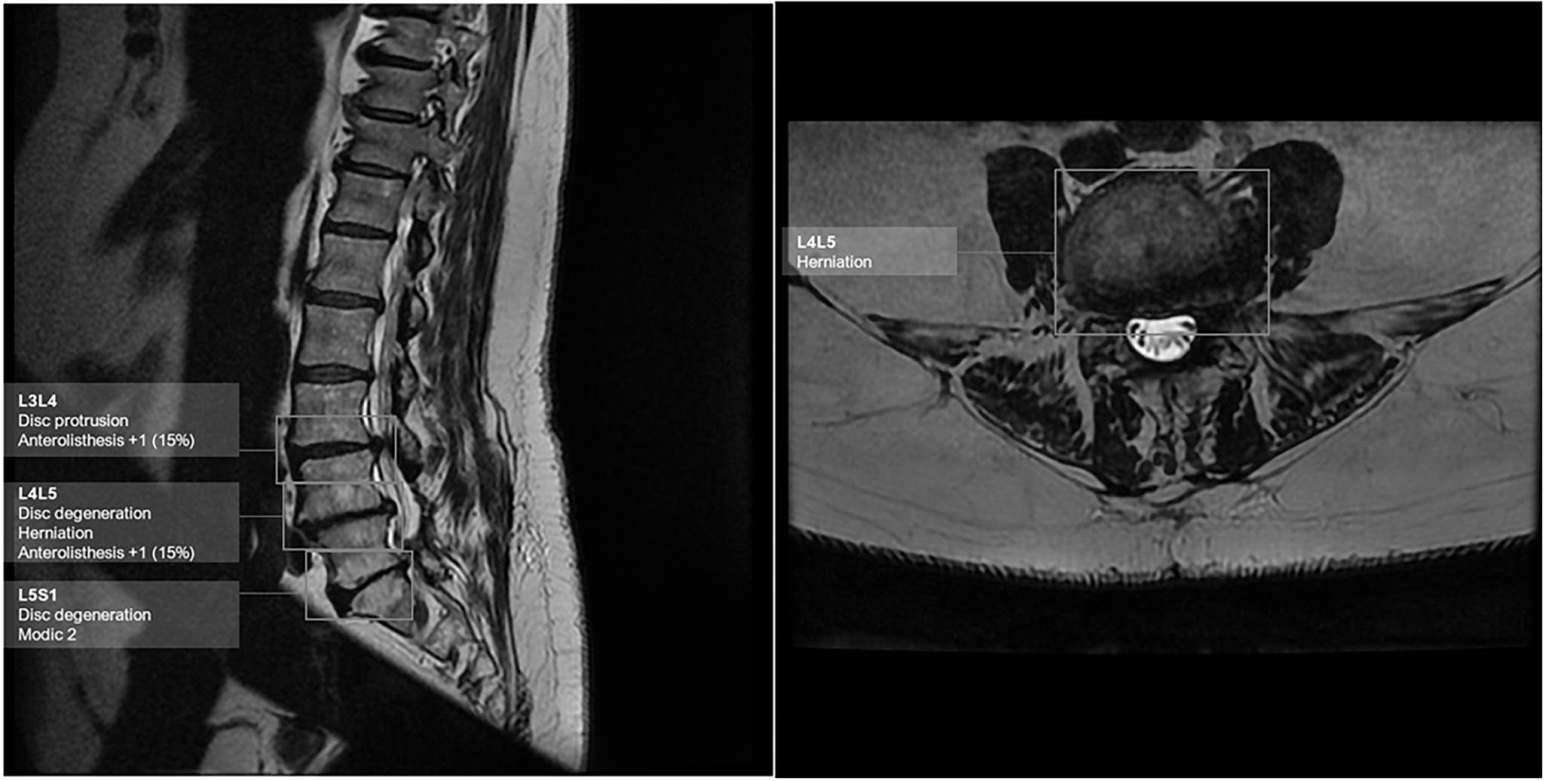
Detection of degenerative disc disease of the lumbar spine with herniated L4-L5 disc, L3-L4 and L4-L5 anterolisthesis, and MODIC 2 vertebral endplates remodelling at L5-S1, using LumbAI software by Caerus medical.

Finally, automated detection of disc herniation on MRI could be a gain of time while allowing a better standardization of the quantification of these protrusions^27^ (Figure 8).

Of note, image interpretation tools may be subject to algorithmic bias, related to the training of AI models predominantly on images from specific populations, or patients scanned with particular equipment or protocols. Such an imbalance may lead to poorer performance when interpreting studies from underrepresented groups, potentially affecting diagnostic accuracy and treatment decisions.

Key takeaway: AI interpretation tools go beyond improved sensitivity and specificity for tasks such as fracture detection or meniscal tear classification. They also help standardize reporting and reduce diagnostic variability across radiologists with different experience levels. In clinical practice, this can narrow performance gaps between general and expert MSK radiologists, directly supporting more equitable patient care.

## LLMs applied to MSK radiology

Another trend in AI applications in the medical field is the use of LLMs for different tasks. The most known include ChatGPT, Llama, Gemini, Claude, DeepSeek, and Grok. Other LLMs were specifically developed for medical purposes, such as MedPalm (Google), with reported better performances for health-related applications compared to the “general” LLMs.^28^ These models often incorporate medical and disease-specific knowledge bases, and are pre-trained on large databases of clinical notes, radiology reports, and biomedical literature. These models are designed to reduce the risk of misinformation in a clinical context and align output more closely with medical guidelines.

Multiple applications of LLMs could be applied to MSK imaging. Recent studies described potential for automated generation of the “impression” section of reports based on findings.^29^ Information of patients is also of upmost importance, with uncommon medical terms in MSK imaging reports. One of the most promising applications of LLMs in MSK imaging is the simplification of imaging reports.^30^ Radiologists often include complex and sometime confusing terminology and detailed anatomical and pathological descriptions in these reports. While these reports are essential for communication with other healthcare professionals, most patients are not familiar with medical terminology. LLMs can generate simplified, patient-friendly summaries while preserving clinically relevant details. This has the potential to improve patient engagement and understanding.^31^

Automated classification of the different MSK disorders based on the description in the reports (eg, Salter-Harris fracture classification in paediatric patients) is another potential useful application of LLMs.^32^ Additionally, this potential could be expanded for soft-tissue and bone tumours classification and risk assessment.

Finally, automated generation of radiographs reports based on AI-based angle measurements, for example in hallux valgus workup, may be another possible application.

Nonetheless, it is important to underline the current limitations. LLMs can sometimes generate plausible sounding but incorrect statements. Therefore, any imaging-related text generated by LLMs must be verified by a radiologist or other qualified medical professionals to ensure accuracy and prevent miscommunication.

Key takeaway: LLMs represent a new layer of AI integration, extending impact from image analysis to language-based tasks. By simplifying radiology reports, automating classification schemes, and generating structured impressions, LLMs can improve patient-physician communication and streamline multidisciplinary workflows. With proper safeguards, these models could become a bridge between radiologists, clinicians, and patients.

## Integration of AI solutions in the clinical workflow

The promise of AI is often expressed in terms of speed and efficiency gains.^33^ In MSK imaging, the total volume of studies can overwhelm radiologists. AI can help shorten reporting times by triaging urgent or complex cases.^34^ If an AI algorithm detects an urgent fracture on X-ray or CT-scan, for example a cervical spine fracture, that study could be escalated to the top of the worklist, ensuring the radiologist addresses critical findings more quickly.^35^

Integration of these AI solutions in the workflow have the potential to streamline the entire process of imaging acquisition and interpretation, with decreased workload for radiologists and improved understandability and standardized care for the patients.

First, the protocol of imaging examinations may be pre-processed by LLMs, and verified by operators (technicians, residents, radiologists).^36^ Then, as previously demonstrated, imaging acquisition may be accelerated with DL reconstructions. Once the acquisition is performed, image interpretation and reporting may also be better standardized with AI tools and LLMs, with probable decreased analysis and reporting time needed per examination.

An example of AI integration into clinical workflow is the implementation of software dedicated to automated fracture detection, which can take various forms. Some institutions automatically run these AI tools for each examination, sometimes automatically triaging positive cases and ensuring that radiologists are alerted of potentially urgent findings. Other institutes favourize on-demand approach, where the radiologist can request an AI analysis of suspicious radiographs. Regardless of the modalities of integration, a growing body of evidence suggests that these tools can enhance efficiency and reduce diagnostic errors.^37^

The importance of the adequate integration of these AI solutions in the workflow of the healthcare professional is one of the current challenges, with often different AI tools needed to perform different tasks. Picture Archiving and Communication Systems (PACS) and Radiology Information Systems (RIS) may not be readily compatible with all these AI tools. Bridging these systems, where the AI tool runs seamlessly on incoming studies and presents results in a user-friendly manner, requires technical solutions such as DICOM-compliant interfaces, standardized imaging protocols, and robust data security frameworks.

In some cases, cloud-based platforms offer an alternative. They can perform the computationally intensive task of AI model inference off-site and return annotated results to local PACS. However, data transfer speeds, data privacy and storage concerns and costs associated with cloud services are additional potential barriers.

The integration of AI solutions is also modulated by regulatory institutions. In many countries, AI software used for diagnostic purposes requires clearance from official agencies (eg, the Food and Drug Administration (FDA) in the United States, the CE marking process in Europe). It is essential that AI models ensure compliance with these regulations and implement robust quality assurance and periodic updates.

Key takeaway: The real-world success of AI depends on its seamless integration into PACS/RIS environments, adherence to regulatory standards, and compatibility with diverse clinical practices. For MSK radiology, carefully designed workflows are essential to ensure that AI reduces reporting time and supports clinical decision-making without creating new inefficiencies or risks and without compromising patient safety.

## Cost-effectiveness of AI in MSK radiology

An important consideration for AI tools in MSK imaging is their economic impact. Solutions most likely to achieve widespread adoption are those that enhance radiologist efficiency while reducing diagnostic errors, thereby offsetting their implementation costs.^38^ Commercial AI software is typically subscription- or license-based, and in some countries these expenses may be passed on, at least in part, to patients through increased examination fees. The actual costs of adoption vary widely, encompassing software licencing, integration with existing PACS/RIS systems, hardware upgrades, and ongoing maintenance and support. Robust data on the average additional expenditure for healthcare organizations remains limited, and further research is needed to clarify the cost-effectiveness of AI in routine MSK imaging practice.

Key takeaway: Long-term adoption of AI in MSK radiology will depend on demonstrating cost-effectiveness. Tools that reduce diagnostic errors, enhance radiologist productivity, and standardize reporting have the best chance of offsetting implementation costs and achieving sustainable clinical value.

## AI integration liability

Among healthcare professionals, radiologists are one of the specialty most subject to malpractice lawsuits related to missed or delayed diagnoses.^39^

As AI becomes more integrated into routine practice, questions arise about how liability is apportioned if AI-driven tools contribute to diagnostic errors.^40^ From a legal point of view, liability generally hinges on whether the standard of care was met. Radiologists are responsible for their interpretative decisions, whether they rely on AI or not.

On the other hand, AI might potentially reduce the risk of malpractice and claims since multiple studies demonstrated the improved accuracy of readers with these tools.^41^^,^^42^ If AI systems can catch missed diagnoses before the signature of final reports, the net effect could contribute to fewer medical-legal disputes.

Additional issues have been raised by the rise of LLMs. These tools may occasionally provide wrong medical recommendations, including in the medical imaging field. However, in practice there is a limited precedent for holding the organizations that deploy or maintain these models liable, in part because many LLMs embed disclaimers or explicitly suggest consulting a medical professional. Moreover, liability typically depends on whether a clinician’s reliance on the tool violated established standards of care, and physicians generally retain ultimate responsibility for the final interpretation and decision.^7^^,^^43^

Vendors and developers of AI tools may also bear some responsibility if the product was marketed with certain performance claims or did not meet regulatory standards. There is a push towards more transparent disclaimers about AI performance metrics and known failure modes. This is particularly important for LLMs, which can generate incorrect or misleading information that sounds highly confident.

Therefore, AI should be introduced and used as an assistive technology to physicians, an advanced “second pair of eyes”, rather than a replacement of the medical expertise.^41^ Institutions adopting AI solutions may also want to consider additional insurance policies or legal frameworks that address AI-related claims. As guidelines and regulations evolve, clear standards for AI verification and validation will be essential in establishing best practices and mitigating legal risks.^7^

Addition challenges include specific billing associated with AI tools, the adaptation of AI to certain populations (eg, older osteoporotic subjects for fracture detection), and the changes in medical education of trainees, which may rely too much on these solutions.

Key takeaway: AI is best positioned as an assistive tool, reinforcing the radiologist’s expertise rather than replacing it. Clear guidelines, transparent performance reporting, and evolving legal frameworks will be crucial to mitigate liability risks and promote safe, responsible adoption of AI in MSK radiology.

## AI in medical education

The integration of AI tools during medical school and throughout the residency is now well established. Students increasingly rely on LLMs for easily accessible knowledge, with very high performances of these models on United States Medical Licensing Examination (USMLE) questions.^44^ AI is also shaping MSK radiology training where numerous automated platforms assist with image interpretation. For example, fracture detection algorithms might reduce trainees’ direct experience in radiograph interpretation. However, with proper pedagogical integration, the decreased burden can instead enhance learning, much as the advent of calculators ultimately strengthened, rather than weakened, students’ mathematical skills.

Key takeaway: Integrating AI into MSK radiology education offers opportunities to enhance, rather than diminish, trainee learning. Properly designed curricula can leverage AI as a supportive tool while preserving critical interpretive skills, preparing the next generation of radiologists for an AI-enabled practice environment.

## Conclusion

In conclusion, AI is rapidly transforming MSK radiology across all stages of the imaging workflow. DL-based reconstruction protocols shorten acquisition times and reduce artefacts, while AI-driven image interpretation enhances diagnostic accuracy, standardizes reporting, and reduces variability between readers. Emerging applications of LLMs extend these benefits beyond image analysis, offering new opportunities for patient-centred reporting and more efficient communication across healthcare teams.

At the same time, successful translation of these technologies into routine practice requires careful consideration of workflow integration, cost-effectiveness, regulatory compliance, and liability. Radiologists must remain actively engaged as final decision-makers, ensuring that AI functions as a powerful assistive tool rather than a substitute for clinical expertise. Furthermore, medical education must evolve to incorporate AI literacy while preserving essential interpretive skills.

Taken together, these developments signal a fundamental shift in MSK radiology towards an AI-enabled environment that is faster, more accurate, and more patient-centred. To fully realize this potential, the field must prioritize not only technical performance but also safe implementation, equitable access, and ongoing education. With these safeguards in place, AI has the potential to enhance radiologists’ capabilities, improve patient outcomes, and reshape the practice of MSK radiology in meaningful and lasting ways.
